# Heart Failure: From Typical Clinical Manifestations to the Surprising Final Diagnosis

**DOI:** 10.7759/cureus.26870

**Published:** 2022-07-14

**Authors:** Maria Margarida Robalo, Inês M Araújo, Rui M Domingues, Marta Viana Pereira, Sofia Esperança

**Affiliations:** 1 Internal Medicine, Hospital de Braga, Braga, PRT; 2 Medical Oncology, Hospital de Braga, Braga, PRT

**Keywords:** cardiac surgery, echocardiography, myxoma, cardiac tumors, heart failure

## Abstract

The authors report a case of an 80-year-old woman with multiple cardiovascular risk factors, with exuberant acute congestive heart failure at admission. Fever, anemia, and an increase in inflammatory parameters were present, with imaging suggesting a respiratory infection as the main reason for decompensation. Empirical antibiotic therapy was instituted, with no clinical improvement even after escalation to broad-spectrum antibiotics and non-invasive ventilation with high support pressures, with no possibility of weaning. Due to maintenance of symptoms, a transthoracic echocardiogram was performed, revealing a large left atrial myxoma, obstructing the mitral valve in diastole. This case illustrates the potential severity of these benign tumors and their ability to mimic symptoms that are often evaluated in the daily life of an internist. The high clinical suspicion led to a diagnosis that was surprising due to its rarity and severity, with the patient being urgently referred for cardiac surgery.

## Introduction

Despite its rarity, with an estimated incidence of 8 to 150 cases per million, myxomas are the most common type of benign primary cardiac tumor [1]. Their clinical spectrum varies regarding size, location, and mobility, with the vast majority developing in the left atrium. The difficulty to detect them correlates with the lack of specific signs and symptoms; from asymptomatic patients to potentially fatal complications [2-4], these benign tumors require an early diagnosis and referral for urgent surgical treatment.

## Case presentation

The authors report the case of an 80-year-old woman, previously independent with regards to daily life activities, with a personal history of arterial hypertension, obesity, dyslipidemia, and hyperuricemia, medicated with diuretic and antihypertensive drugs.

She presented with dyspnea on exertion for about three weeks, which evolved to dyspnea at rest, orthopnea, and paroxysmal nocturnal dyspnea. In association with this escalation of symptoms, she described intermittent fever for four days and a productive cough. On physical examination, there were clear signs of respiratory distress, with polypnea at rest, supraclavicular retraction, and use of accessory muscles. Her heart rate was 100 bpm, blood pressure 100/55 mmHg, peripheral oxygen saturation 85% (FiO2 0.21) and body temperature was normal. She had cold extremities but no signs of poor circulation, jugular vein distention, hypophonic sounds, absence of heart murmurs or gallop rhythms at cardiac auscultation, and pulmonary auscultation with bilateral crackles, without significant peripheral edema. Given the respiratory effort and peripheral desaturation, the patient immediately started non-invasive mechanical ventilation (NIV) with high support pressures and supplemental oxygen therapy, titrated up to 15L/min.

The initial study showed a type 1 respiratory failure and mild respiratory alkalemia on arterial blood gas analysis, as well as sinus tachycardia on the electrocardiogram, with no signs of acute ischemia. Blood analysis showed normocytic normochromic anemia, an increased C-reactive protein level, leukocytosis with neutrophilia, thrombocytosis, NT-proBNP over 30000 pg/mL, with negative markers for myocardial necrosis, as well as an increased plasma creatinine and urea (Table 1). Pneumococcus urine antigen was negative and the remaining results were unremarkable.

**Table 1:** Abnormal laboratory findings on admission WBC, white blood cells; PLT, platelets; CRP, C-reactive protein.

Parameter	Values	Reference values
Hemoglobin, g/dL	9.2	12.1 - 15.1
WBC, 10^9^ cells/L	26	4.5 - 11.0
PLT, 10^9^ cells/L	501	150 - 400
Serum creatinine, mg/dL	2.5	0.7 - 1.3
Serum urea, mg/dL	100	6 - 24
CRP, mg/L	65	8 - 10
NT-proBNP, pg/mL	34524	< 450

**Figure 1:**
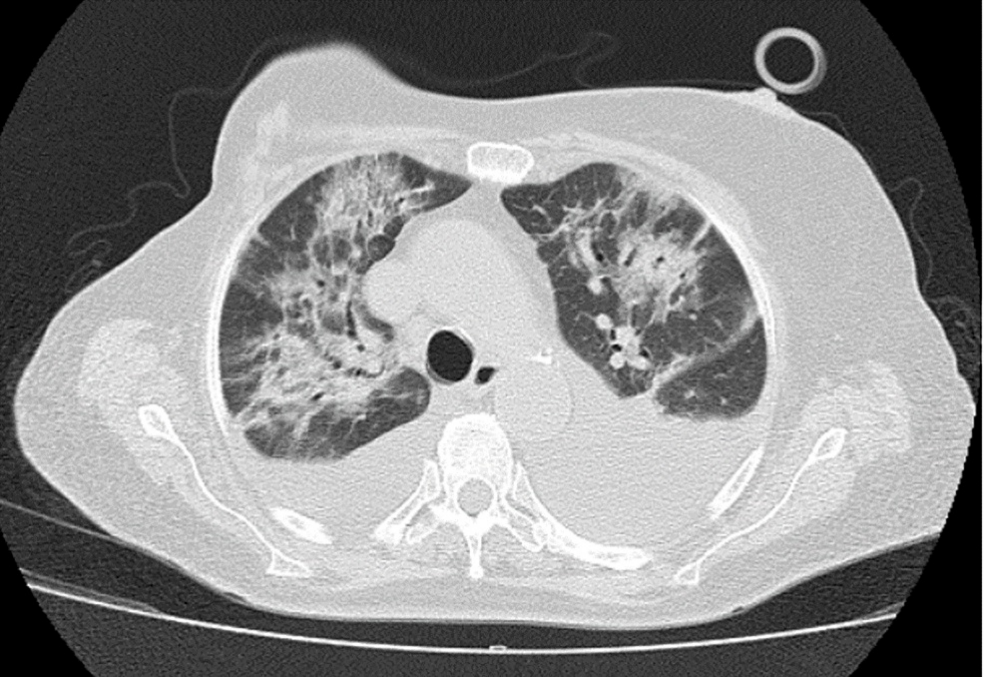
Chest-CT scan with bilateral pleural effusion and sparse ground-glass areas.

A chest computed tomography (CT) scan, limited by the non-administration of contrast, showed "cardiomegaly, bilateral pleural effusion and dispersed ground-glass areas, compatible with an infectious process" (Figure 1).

Thus, the patient was admitted to the hospital with the diagnosis of acute congestive heart failure, most probably decompensated by community-acquired pneumonia, with a severe type 1 respiratory failure, dependent on NIV and supplemental oxygen therapy. Empirical antibiotic therapy with ceftriaxone and clarithromycin was instituted in this context.

A complementary investigation confirmed normocytic normochromic anemia with no iron deficiency, nitrogen waste retention with an improving prerenal acute kidney injury, normal serum ionogram, descending inflammatory markers, and no changes in thyroid function or serum protein electrophoresis. Blood and sputum cultures were negative, as well as the respiratory viral panel. Despite optimized diuretic therapy and ongoing antibiotic treatment, after four days of admission, the patient maintained severe dyspnea at rest and type 1 respiratory failure in progression (PaO2/FiO2 ratio <200 mmHg), still dependent on NIV with high support pressures (inspiratory positive airway pressure: IPAP 18 cmH2O, expiratory positive airway pressure: EPAP 10 cmH2O), with fast reduction of peripheral oxygen desaturation to 70% with its suspension for short periods.

**Figure 2:**
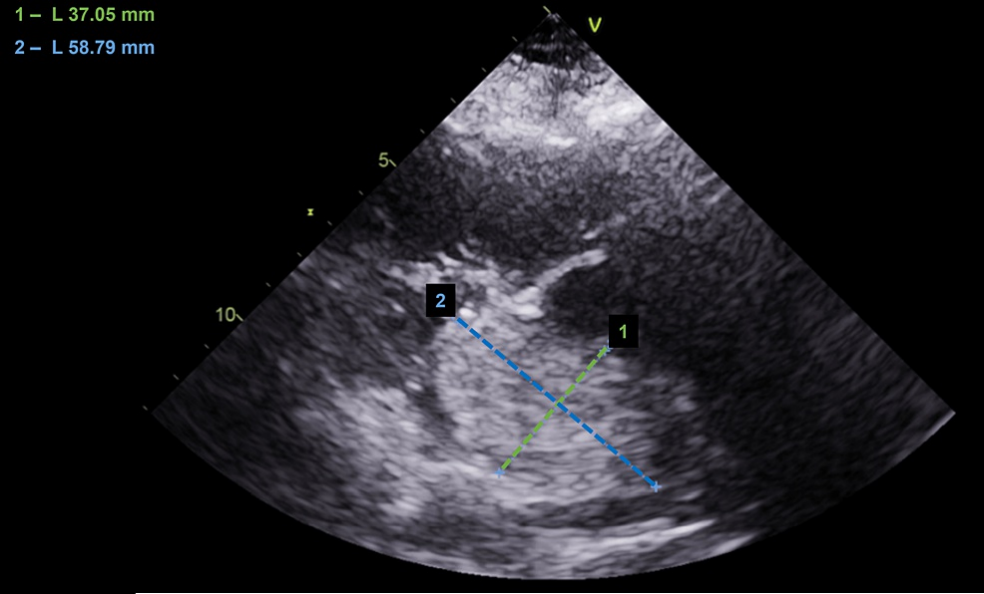
Transthoracic echocardiogram showing the left atrium filled with a mass measuring about 60x40 mm.

**Figure 3:**
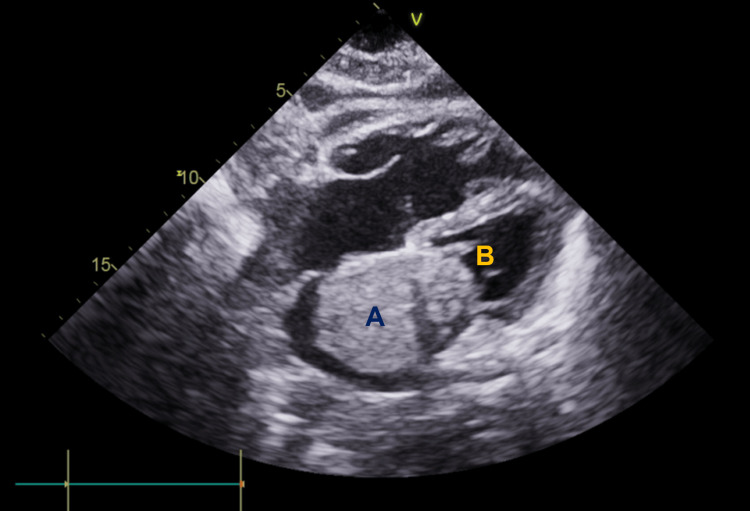
Transthoracic echocardiogram with a clear intracardiac mass (A) obstructing the mitral valve (B) in diastole.

For this reason, a transthoracic echocardiogram was requested, revealing a moderate dilation of the right cavities and the left atrium, the latter filled with a mass measuring about 60x40 mm, obstructing the mitral valve in diastole (Figures 2-3). Global left ventricular systolic function was preserved and an abnormal movement of the interventricular septum suggested a pressure overload. Pulmonary artery systolic pressure (PASP) was 80 mmHg, indicating pulmonary hypertension due to left ventricular diastolic dysfunction.

Therefore, given the suspected diagnosis, absence of active infection, and analytical improvement, the patient was transferred to the Cardiac Surgery department and submitted to an urgent excision of the auricular mass, without any intraoperative complications. Its histopathological examination confirmed a “hypocellular neoplasm with myxoid stroma, with the presence of erythrocyte extravasation and fibrin, with no signs of malignancy, measuring about 70 mm in diameter and 72 g in weight, compatible with an auricular myxoma”.

About a week after surgery, the patient had no signs of respiratory distress, tolerating well the NIV weaning process. NT-proBNP improved to 5200 pg/mL and a transthoracic echocardiogram on day 10 after surgery showed a preserved systolic function and a PASP of 53 mmHg, with no further pharmacological treatment.

On day 15 after surgery, the patient developed hospital-acquired pneumonia complicated with septic shock, requiring reintubation and vasopressor support. Meropenem was immediately started. Blood cultures were negative but the sputum sample tested positive for *Serratia marcescens* and *Klebsiella pneumoniae* carbapenemase-producing isolates. She started amikacin in association with meropenem according to the antibiogram profile but there was no response to the instituted therapy and the patient died 11 days later.

## Discussion

Primary cardiac tumors are rare, with an incidence that varies between 0.0017 and 0.03%, with secondary cardiac lesions having an incidence 30 times higher. Approximately 75% are benign tumors, with myxomas being the most frequent [2,5], with a higher prevalence in women (2:1) between 50 and 60 years of age. Atrial myxomas are generally sporadic, but they may more rarely occur in the familial form, in younger individuals, with an autosomal dominant hereditary pattern, usually associated with other lesions (eg. Carney complex) [2, 4-6].

Its clinical manifestations are diverse, ranging from incidentally detected masses in asymptomatic individuals to symptoms related to cardiac obstruction, systemic embolization mostly to the central nervous system, direct invasion of the myocardium or adjacent lung parenchyma, or even constitutional signs and symptoms [2, 4-6]. The latter is justified by the release of pro-inflammatory factors by the tumor and includes fever, weight loss, and myalgias, among others, also explaining the possible increase in CRP, leukocytosis, thrombocytosis/thrombocytopenia and anemia [2-5].

In symptomatic and large left atrial myxomas that cause a barrier to intracardiac circulation, it is understandable the development of typical symptoms and signs of heart failure, namely dyspnea, orthopnea, paroxysmal nocturnal dyspnea, pulmonary edema, and fatigue, with the vast majority of patients having pulmonary hypertension at the time of diagnosis [2,4-7].

Therefore, the difficulty to reach the final diagnosis in this particular case is understandable, given the frequency of patients with the aforementioned symptoms admitted to an Internal Medicine ward: severe congestive heart failure (NYHA class IV) in an elderly patient with multiple cardiovascular risk factors, systemic arterial hypotension, and prerenal acute kidney injury. The relief of symptoms and high dependency on NIV with high support pressures, due to the consequent reduction in preload, led to the suspicion of a primary cardiac cause for the signs and symptoms that persisted.

Transthoracic echocardiography remains the first-line test, allowing a presumptive diagnosis of myxoma and summary characterization of the tumor (location, size, mobility, and impact on cardiac function). It can be followed by transesophageal echocardiography for better morphological characterization. Cardiac CT scan and magnetic resonance imaging (MRI) are non-invasive alternative tests that can also identify the presence of a cardiac mass. In fact, cardiac MRI can provide data that can further clarify the diagnosis, performing better than echocardiography at determining the nature of cardiac lesions and differentiating myxomas from other cardiac masses. Definitive diagnosis requires tumor excision and anatomopathological confirmation [4-6].

Surgical treatment is crucial, usually curative, and in most cases, associated with a fast regression of clinical and laboratory findings. Consequently, cardiac myxomas are a treatable cause of heart failure [2, 4-6]. Nonetheless, despite being a benign tumor, its morphological characteristics and location can lead to potentially fatal cardiovascular complications, and recovery will always depend on the patient's functional reserve.

## Conclusions

Although most patients with cardiac myxomas lack specific symptoms that mimic other frequent diseases, under high suspicion the diagnosis can be easily made using non-invasive procedures. Surgical resection remains the mainstay of treatment, preventing dreaded complications, with a rapid resolution of the clinical manifestations, generally with an excellent long-term prognosis.
